# Protein-Based Hydrogels and Their Biomedical Applications

**DOI:** 10.3390/molecules28134988

**Published:** 2023-06-25

**Authors:** Kok Zhi Lee, Juya Jeon, Bojing Jiang, Shri Venkatesh Subramani, Jingyao Li, Fuzhong Zhang

**Affiliations:** 1 Department of Energy, Environmental & Chemical Engineering, Washington University in St. Louis, One Brookings Drive, Saint Louis, MI 63130, USA; kokzhi@wustl.edu (K.Z.L.); jjuya@wustl.edu (J.J.); bojing.jiang@wustl.edu (B.J.); s.shrivenkatesh@wustl.edu (S.V.S.); jingyao.li@wustl.edu (J.L.); 2 Institute of Materials Science and Engineering, Washington University in St. Louis, One Brookings Drive, Saint Louis, MI 63130, USA; 3 Division of Biological & Biomedical Sciences, Washington University in St. Louis, One Brookings Drive, Saint Louis, MI 63130, USA

**Keywords:** hydrogels, recombinant proteins, protein hydrogel, microbial expression, protein polymers, synthetic biology

## Abstract

Hydrogels made from proteins are attractive materials for diverse medical applications, as they are biocompatible, biodegradable, and amenable to chemical and biological modifications. Recent advances in protein engineering, synthetic biology, and material science have enabled the fine-tuning of protein sequences, hydrogel structures, and hydrogel mechanical properties, allowing for a broad range of biomedical applications using protein hydrogels. This article reviews recent progresses on protein hydrogels with special focus on those made of microbially produced proteins. We discuss different hydrogel formation strategies and their associated hydrogel properties. We also review various biomedical applications, categorized by the origin of protein sequences. Lastly, current challenges and future opportunities in engineering protein-based hydrogels are discussed. We hope this review will inspire new ideas in material innovation, leading to advanced protein hydrogels with desirable properties for a wide range of biomedical applications.

## 1. Introduction

Hydrogels are swollen three-dimensional materials that absorb at least 10% water (by weight or volume) in their polymer network [1]. Hydrogels have diverse applications in tissue engineering [2], regenerative medicine [2], drug release [3], scaffolding, and adhesives [4]. Hydrogels are usually made of soft synthetic polymers or biopolymers, with the latter type being biodegradable, biocompatible, and bioabsorbable. Comparing different types of biopolymers, such as polysaccharides, DNAs, proteins, and lipids, proteins can fold into diverse structures and display a greater range of chemical and biological functions and thus are attractive hydrogel materials suitable for a wide range of applications, particularly for biomedical engineering. In this review, we discuss hydrogels made of proteins.

**Figure 1:**
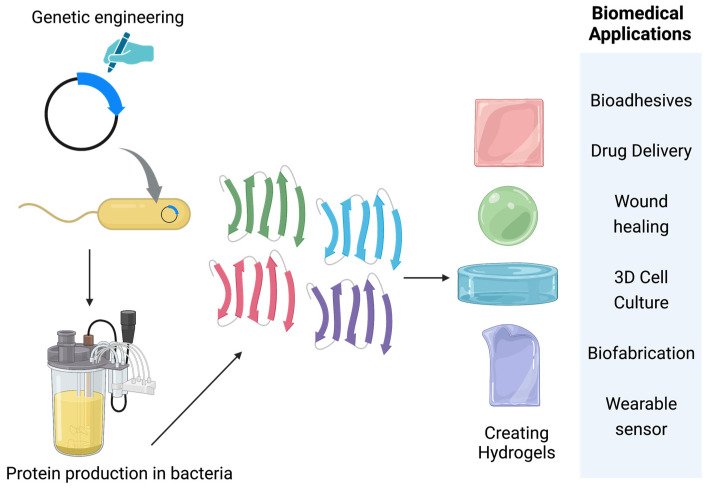
Overview of protein-based hydrogel applications.

Protein-based hydrogels can be further divided into natural protein hydrogels and synthetic protein hydrogels. Natural protein hydrogels, such as silk and collagen, have been extensively studies [5,6]. These hydrogels have good biocompatibility and are widely used for tissue engineering applications [5,6]. However, naturally isolated protein often has large quality variations depending on material source that may result in unreliable material properties. Furthermore, hydrogel properties are often confined by the properties of these natural proteins, thus limiting their applications [5,7]. On the contrary, synthetic proteins produced from engineered microbial hosts circumvent these limitations. As synthetic DNA technology becomes mature, all natural proteins can be potentially encoded with synthetic DNA to facilitate high titer expression in fast-growing microbial hosts on large scales. Modern DNA assembly techniques such as Gibson [8] and Golden-Gate DNA assembly [9] allow for rapid construction of multiple synthetic DNA fragments to code complex proteins. Scientists can further modify protein sequences to create hydrogels with programmable properties for different applications (Figure 1) [4]. In this review, we discuss protein hydrogels with special focus on those made of microbially produced proteins. We first describe hydrogel formation mechanisms and then discuss hydrogels from different sources and biomedical applications. Finally, we discuss current challenges and future directions.

## 2. Hydrogels Crosslinking Strategies

**Figure 2:**
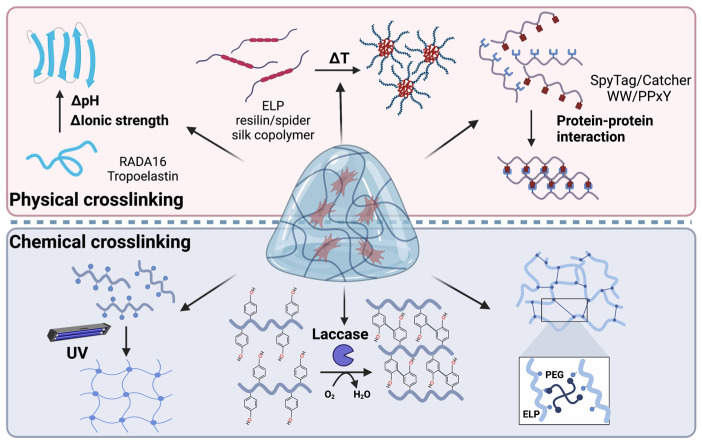
Different methods for the fabrication of protein-based hydrogels.

Hydrogels can be prepared from proteins through various physically crosslinked mechanisms such as non-covalent physical interactions or chemical crosslinking using crosslinker or protein ligation reactions (Figure 2).

### 2.1. Physically Crosslinked Protein Hydrogel

Physically crosslinked protein hydrogels are formed by polymer chain entanglements or through non-covalent physical interactions between polymer chains [10]. Proteins can interact with each other via electrostatic interaction, hydrogen bonding, Van der Waals interaction, and hydrophobic effects. Some disordered proteins use a combination of these interactions to form networks [11]. However, random interactions between disordered proteins often lead to protein aggregation, precipitation, coacervation or hydrogels that are extremely soft. On the other hand, some proteins fold into defined secondary or tertiary structures, allowing them to form three-dimensional (3D) interacting networks at the macroscopic scale and to entrap water, thus forming hydrogels [10,12,13,14,15,16]. Such physically crosslinked protein hydrogels avoid any toxic crosslinkers and can have attractive mechanical properties, making them suitable for a wide range of biomedical applications [12]. Here, we discuss strategies that trigger protein self-assembly and hydrogel formation.

#### 2.1.1. pH and Ion-Induced Protein Hydrogels

Protein self-assembly and hydrogel formation can be triggered by changing pH or ionic strength. In this case, proteins are often highly charged, and their electrostatic repulsion prevents their assembly in low ionic strength buffers. Upon changing buffer pH to neutralize their charges or adding salt to shield their electrostatic repulsion, protein chains can come close and self-assemble to form hydrogels. One example is the designed RADA16 peptide (RADARADARADARADA) [17]. When electrostatic repulsion is shielded, the repetition of hydrophilic and hydrophobic residues in RADA16 folds into a β-strand, allowing multiple peptides to form a β-sheet that further packs together through hydrophobic or other interactions to form 3D hydrogels. Many other β-sheet-forming peptides or proteins can form hydrogels in this manner [18]. Ionic strength and pH needed to induce hydrogel formation can be tuned by changing the number of charged residues in these proteins [16]. Such hydrogels are often weak due to their weak inter-sheet interactions. One effective strategy to substantially improve hydrogel mechanical properties is to fuse β-sheet-forming proteins with self-interacting domains. Kim et al. genetically fused a self-interacting mussel foot protein (Mfp) to β-sheet-forming amyloid-silk repeats, where multiple amyloid peptides are fused with flexible linkers from a silk protein. It was shown that the amyloid-silk 8xKLV (fusion of eight repeats of KLVFFAE and silk) hydrogel is brittle, breaking at an ultimate strain of 3%. In contrast, the fusion of Mfp to 8xKLV enhanced hydrogel strain by 100-fold, reaching 300%, with an ultimate tensile strength of 1.0 ± 0.5 MPa and 1 Mpa elastic modulus [4].

pH and ions can also induce hydrogel formation from α-helical proteins. Tropoelastin, a common elastin in the human body, forms an α-helical polyproline type II-like structure under an alkaline buffer with pH above 10 [19]. The self-assembly process is initiated by changes in ionic strength, initiating coacervate formation. This assembly brings lysines of intermolecular tropoelastin close together to facilitate crosslinking reaction via lysyl oxidase. The mechanical properties of these hydrogels can be adjusted by modifying the pH, protein concentration, and ionic strength of the solution, with their elastic moduli ranging from 8 to 20,000 kPa [20,21,22]. These hydrogels offer a well-defined, fully re-absorbable extracellular matrix (ECM) analogue that can be used to investigate ECM’s effects on cell behavior [23].

#### 2.1.2. Temperature-Induced Hydrogel Formation

Temperature can change protein interactions and trigger hydrogel formation. Hydrogels that can form rapidly near body temperature are particularly attractive because they can be used as injectable materials in wound healing, cell therapy, and drug delivery [24]. Elastin-like proteins (ELPs) are the best-known temperature-responsive proteins. ELPs contain repetitive VPGXG (X can be any amino acids except for proline) sequences and can undergo reversible phase transitions at different temperatures. While creating hydrogels using ELPs is challenging due to their high hydrophobicity and tendency to aggregate heterogeneously, ELPs can be modified by incorporating hydrophilic regions or structural domains such as silk to create temperature-responsive hydrogels [25,26]. The resulting hydrogels exhibit good mechanical properties and can be fine-tuned by adjusting the crosslinking density [25,26].

Resilin, an elastomeric protein found in insects, exhibits remarkable mechanical properties such as the ability to recoil [23]. Resilin-inspired copolymer hydrogels have been developed by genetic fusion of resilin-like polypeptides with thermo-responsive polymers such as spider silk carboxyl-terminal (CT) domain [23]. The resilin and spider silk CT copolymer led to improved mechanical properties, and a shift in gelation temperature to a range that is more physiologically relevant than hydrogel only consisted of silk CT protein. Additionally, Luo et al. observed that the copolymer hydrogel released the drug molecule rhodamine B in pH 7.2 PBS by 66%, due to the pH-responsiveness of the resilin component. These findings suggest that the copolymer hydrogel could have potential as a multi-responsive material in various biomedical applications [23,24,27,28].

#### 2.1.3. Protein–Protein Interaction-Induced Assembly of Hydrogels

One drawback associated with the above-discussed pH-, ion-, or temperature-induced hydrogels is that their properties are susceptible to alterations in environmental conditions such as pH and temperature changes [29]. One strategy to address this is to form hydrogels based on specific protein–protein interactions [10]. One commonly used protein–protein interaction pair is the WW domain with proline-rich proteins [30]. The WW domain refers to the protein consisting of 40 amino acids with two signature tryptophan residues. Wong Po Foo et al. developed the first mixing-induced two-component protein hydrogel using a pair of WW–Proline-rich peptide (PPxY) domains derived from the p53 binding protein. In their approach, multimers of the WW domain and protein-rich domains were connected by flexible hydrophilic linkers [31]. This design facilitated the binding of WW and PY ligand domains and contributed to the viscoelasticity of the hydrogel. Additionally, the hydrogels exhibited shear-thinning and self-healing properties, making them suitable for a variety of cell-encapsulation applications [31].

### 2.2. Chemically Crosslinked Protein Hydrogel

Another common strategy to prepare protein hydrogel is chemical crosslinking, either using multi-functional small-molecule crosslinkers or through incorporation of specialized functional groups into proteins for covalent linking. Chemically crosslinked hydrogels are generally more stable against environmental perturbation and degradation than physically crosslinked hydrogels [32,33,34]. Here, we summarize a few commonly used chemical crosslinking methods.

#### 2.2.1. Light-Controlled Protein Hydrogel Formation

Proteins modified with unsaturated (e.g., methacrylate) or photosensitive (e.g., azobenzene) functional groups can be crosslinked by radical-based reactions to form a 3D network [32,35]. The production of free radicals can occur through several methods, including heating, ultraviolet radiation, high energy radiation, electrolysis, and plasma initiation [34,36,37]. For example, lysine residues in silk protein have been reacted with glycidyl methacrylate, which enables silk proteins to be crosslinked by UV light with a photo-initiator, generating hydrogels with 10–15 kPa elastic moduli [38]. Photo-crosslinking allows for rapid hydrogel formation at mild temperatures with a controlled degree of crosslinking. The precise spatial control of photo-initiated crosslinking also allows hydrogels to be made from 3D printing [38]. Further, protein hydrogels can be formed using light-switchable protein–protein interactions. Narayan and colleagues created photo-responsive hydrogels using the light-switchable CarH protein [33]. In the presence of adenosylcobalamin (AdoB12), the C-terminal domain of CarH binds to AdoB12 and forms tetramer in the dark but becomes monomeric when exposed to white light. By fusing a silk-elastin-like protein (SELP) to CarH, the resulting SELP-CarH protein formed hydrogels in the dark due to CarH oligomerization as well as dissolved in solution when exposed to white light. Additionally, Guo et al. created methacrylated ELPs (ELP-MA) by modifying lysine-rich ELPs with methacrylate groups, which enabled photo-crosslinking of the ELP-hydrogel [39]. By changing the degree of methacrylation and by using the temperature-induced phase transition of ELPs, the mechanical properties of the ELP-MA hydrogel could be adjusted. The ELP-MA hydrogel has potential applications in biomedical fields from its unique characteristic of regulating light transmission and elastic adhesiveness [39].

#### 2.2.2. Chemical Crosslinker-Based Protein Hydrogels

Multi-functional chemical crosslinkers are commonly used to crosslink proteins and to form hydrogels. Multiple functional groups, such as amine, thiol, and carboxyl groups, can be used to react with a crosslinker. Crosslinking reactions randomly occur on any residue that can react with the crosslinker, which may affect hydrogel properties. To solve this issue, unique residues are often introduced to proteins for site-specific crosslinking. For instance, cysteine residues, which do not commonly exist is ELP, were added to ELP by genetic engineering to enable thiol–maleimide conjugation between ELP and maleimide-containing polymers (e.g., hyaluronic acid) or crosslinkers (e.g., polyethylene glycol, glutaraldehyde, maleimide, N-hydroxysuccinimide esters), thus forming hydrogels [40,41,42,43,44]. Such chemically crosslinked ELP hydrogels exhibit elastic behavior and can withstand axial deformation, stress, and mechanical corrosion [45]. Additionally, the potential cytotoxicity from unreacted crosslinkers of these materials requires further studies [32,46].

#### 2.2.3. Enzymatic Crosslinked Hydrogels

Hydrogels can be created through various enzyme-catalyzed reactions between specific peptide sequences or peptide–ligands. These enzymatic reactions are highly specific and can be used to engineer hydrogels of complex structures using multiple different enzymes [47]. Additionally, gelation kinetics can be controlled by adjusting the enzyme concentrations and activity [48]. One example is crosslinking of ELP by transglutaminase, an enzyme that catalyzes the conjugation between lysine and glutamine sidechains [49]. Another commonly used enzyme for crosslinking is laccase, a multicopper oxidase that oxidizes phenolic compounds [50]. Laccase has been used to crosslink SELPs, resulting in rapid gelation and excellent biocompatibility [51]. Additionally, a SpyTag/Catcher-catalyzed ligation reaction was used to crosslink ELP fused with super uranyl binding proteins (SUP) and molybdate/chromate-binding proteins (ModA), forming hydrogels for metal sequestration [52]. While chemical crosslinkers and photo-initiators may induce cytotoxicity of the formed gels, enzyme-crosslinked hydrogels are often less toxic and are more biocompatible [53]. The advantages and disadvantages of the above-mentioned hydrogels are summarized in Table 1.

## 3. Hydrogels Made from Different Protein Sources and Their Biomedical Applications

**Figure 3:**
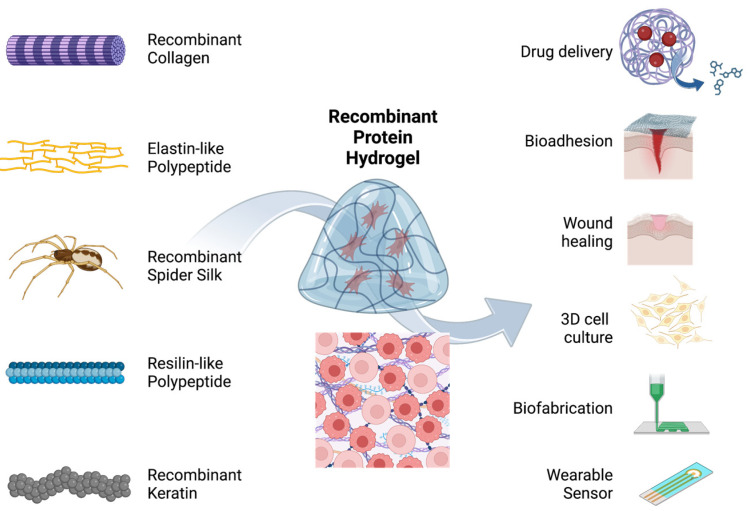
Summary of the applications of microbially produced protein hydrogels. Recombinant or engineered collagen, elastin-like polypeptide, spider silk protein, resilin-like polypeptide, and keratin have been engineered to create hydrogels that were used for bioadhesion, drug delivery, wound healing, 3D cell culture, biofabrication, and wearable sensors.

Due to their biocompatility and tunable mechanical, chemical, and biological properties, protein hydrogels are suitable for biomedical applications, especially in tissue engineering where scaffold or artificial ECMs are needed to regenerate damaged tissues. Specific protein hydrogel applications include bioadhesives for repair, drug delivery, wound healing, 3D cell culture, tissue and neuron regeneration, and biofabrication. In this section, we discuss hydrogels made from different sources, their associated properties, and their biomedical applications (Figure 3 and Table 2).

### 3.1. Hydrogels Made of Natural Proteins

Protein hydrogels were initially prepared from naturally isolated proteins. Diverse hydrogels for scaffolding, drug delivery, and bioadhesion have been developed using animal-derived proteins, including silk, casein, and mussel foot protein [18,56,57,59]. However, the sourcing of these proteins from animal-derived sources poses certain drawbacks, including immunogenicity and batch-to-batch variation on protein qualities (molecular weight, composition, modification, etc.).

### 3.2. Hydrogels Made of Microbially Synthesized Proteins

Microbially synthesized recombinant proteins allow for fine-tuning of hydrogel properties by changing protein sequences and thus can meet more specific requirements of various biomedical applications than natural-protein hydrogels. Here, we discuss hydrogels made from microbially synthesized recombinant proteins of various sequence origins.

#### 3.2.1. Strategies for Microbial Synthesis of Material Proteins

Hydrogels made of recombinant proteins used to be limited by low protein yield and high protein-production costs. Unlike typical water-soluble proteins and enzymes, proteins used for hydrogels are often repetitive, having high molecular weight and biased amino acid compositions. These protein sequence feathers make them difficult to express in microbial hosts, particularly at high levels for sufficient hydrogel fabrication. The yield of highly repetitive material proteins generally decreases with increasing molecular weight due to factors such as DNA/mRNA instability, depletion of heavily used tRNAs, and premature translation termination [47]. Various strategies have been developed to overexpress high-molecular-weight highly repetitive material proteins including codon optimization [60,61] and post-translational ligation of low-molecular-weight protein monomers using split-intein [62,63,64,65,66]. When combining these strategies together with high-cell density fed-batch fermentation technology, highly repetitive material proteins have been recently produced in very high titers ranging from 8 to 30 grams per liter [67,68].

#### 3.2.2. Microbially Synthesize Proteins of Different Origins and Their Applications

Collagen. Collagen is a natural protein that provides structural support to tissues, contributing to their strength, flexibility, elasticity (0.0022–250 kPa elastic modulus), viscoelasticity, and compressibility [69,70,71,72]. Nevertheless, microbes lack complex protein post-translational modifications as seen in collagen; thus, only types I, II, and III recombinant human collagen can be produced from microbial systems [73]. Recombinant collagens display lower inflammatory responses than animal-derived collagens [74] and exhibit the same pH-driven gelation process as tissue-derived collagen [74,75]. Furthermore, recombinant human collagens can provide structural support and strength to tissues, making them attractive for bone tissue engineering [76,77,78]. Studies have demonstrated the ability of recombinant human collagen types I and III to support in vitro epithelium and nerve overgrowth, leading to potential use for corneal substitution and stromal regeneration [79,80,81,82,83]. Furthermore, a combination of recombinant types I and III collagen is promising in the treatment of myocardial infarction [84].

Elastin. Native elastin is insoluble in water and requires harsh chemical treatments for extraction from tissues [85]. However, with the advent of recombinant technology, ELPs can now be synthesized to mimic the physical and chemical properties of native elastin. The use of microbial protein expression systems enables convenient large-scale production of ELPs. Furthermore, their degradation rates, mechanical strength, and cell adhesion can be fine-tuned at the gene level to meet specific tissue engineering requirements [86]. ELPs have a unique property of undergoing a reversible sol–gel transition in response to changes in temperature or other stimuli, which allows them to form hydrogels in situ under physiological conditions. ELP hydrogels have been used to encapsulate cells for cell therapy. For example, ELP was genetically fused to the C-terminal adenosylcobalamin binding domain of photoreceptor C (CarH_C_), whose structure undergoes sol–gel transition upon exposure to white light. The resulting hydrogel was used to encapsulate NIH/3T3 fibroblasts and mesenchymal stem cells (MSCs), which can be released upon white light irradiation [87,88]. These hydrogels can serve as an in vitro platform for 3D cell culture, for studying cell–cell and cell–matrix interactions, and for understanding cell signaling pathways [87,88].

Silk proteins. Spider silk is a highly intriguing natural material, known for its exceptional combination of high tensile strength and high toughness, rarely seen in other types of materials. It is of particular interest in the biomedical field due to its biocompatibility, nontoxicity, slow degradation rate, non-immunogenicity, and elastic properties [89]. Hydrogels made of regenerative silk proteins have been extensively explored [5,90]. While useful and somewhat tunable, their properties are confined by natural silk proteins. Recombinant silk proteins allow for a wider range of hydrogel properties and even new functions to be explored. Schacht et al. added the cell-adhesion RGD motif to a recombinant silk protein, allowing cells to adhere to the resulting silk hydrogels [91]. Koh et al. replaced the repetitive region of spider silk protein with suckerin teeth peptides and incorporated the RGD motif. The resulting fusion protein underwent thermal gelation and exhibited skin cell adhesion and proliferation, making it an attractive scaffold for chronic wound healing [92].

Resilin. Resilin is an intrinsically disordered protein known for its remarkable elasticity, resilience, and fatigue lifetime. It can be found in the cuticles of many insects. Kam et al. leveraged the 6% tyrosine residues in resilin to facilitate [Ru(bpy)_3_]^2+^-mediated photochemical crosslinking, enabling the formation of dityrosine bonds and the generation of a thermoset hydrogel [57]. They also employed this approach to produce cell-laden 3D prints through multiphoton adsorption polymerization [57]. Similarly, Hu et al. applied this strategy to crosslink a reduced graphene oxide-resilin conjugate, resulting in an electronconductive hybrid hydrogel. This hydrogel has been demonstrated to be a flexible wearable sensor for human activities [93]. Kiick and colleagues replaced tyrosine with either phenylalanine or methionine and used [tris(hydroxymethyl)phosphino] propionic acid (THPP) to crosslink the protein into hydrogels [58]. Subsequently, they demonstrated the viability of encapsulating bone-marrow-derived human mesenchymal stem cells (hMSCs) and studied bioactivity after incorporating various cell-binding domains into the hydrogel, including RGD, heparin-binding domains, and matrix metalloproteinase (MMP)-sensitive domains [94].

Keratin. Keratin is a tough, durable fibrous protein that is the primary structural component of hair, nails, feathers, hooves, horns, and claws in vertebrates. Natural sources of keratin have been extensively studied for their potential applications in hemostasis, regeneration, drug delivery, and cell culture [95]. Recently, Hao et al. synthesized soluble recombinant human type I hair keratin 37 and type II hair keratin 81, which were combined with carboxymethyl cellulose to form a hydrogel [96]. This hydrogel demonstrated stronger effects on bleeding cessation, cell migration, and proliferation compared to naturally extracted keratins, suggesting potential applications in biomedical engineering and regenerative medicine.

#### 3.2.3. Multi-Functional Hydrogels from Different Sources

Multiple proteins of different sequence origins can be genetically fused together to create multi-functional proteins and thus hydrogels. For example, a hybrid protein consisting of sequences from spider silk, amyloid, and Mfp were created [4]. The resulting hydrogel contains large amount of β-crystals formed from amyloid sequences, displays high stretchability and toughness benefited from silk protein, and is adhesive to a wide range of surfaces even underwater due to the presence of the Mfp sequence [4]. Similarly, by fusing the light-switchable CarH protein with SELP, the resulting hydrogel shows elastic and stiff mechanical properties with light-responsive behavior [33].

**Table 2:** Biomedical applications of protein-based hydrogels.

Application	Protein	Desirable Properties	References
Bioadhesion	Mussel-foot protein	- forms strong interactions with target tissues	[62]
Drug delivery	Elastin-like polypeptideKeratin	- tunable degradation profile - controlled release - tunable to respond to different pH, ionic strength, and temperature	[97,98]
Wound healing	Suckerin–spider silkKeratin	- biocompatible - slow degradation - able to encapsulate drug or growth factors - reduce inflammation - stabilize homeostasis	[92,99]
3D cell culture	Spider silkElastin–collagenKeratin	- allows cell adhesion - has similar mechanical properties to the target tissue	[91,100,101]
Tissue regeneration	CollagenKeratin–fibrinogen	- has similar mechanical properties to the target tissue - attracts cell migration - promotes cell differentiation	[77,83,84]
Biofabrication	Spider silkCollagen	- biocompatible with tissue of interest - allows cell adhesion - promotes cell growth - can be modified to tune cellular interaction	[80,91]
Wearable sensor	Resilin	- elastic - extensibility - conjugates with chemical to have electromechanical properties	[93]

## 4. Current Challenges and Future Directions

**Figure 4:**
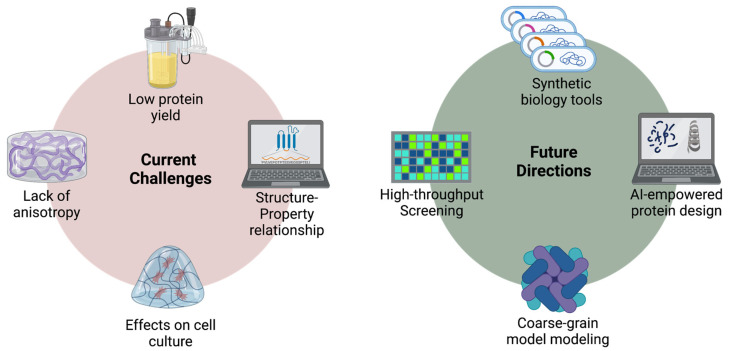
Current challenges and future directions of protein-based hydrogel materials.

Microbially synthesized, protein-based hydrogels exhibit several advantages such as high biocompatibility and biodegradability, tunable mechanical properties, well-defined composition, and homogeneous macroscale structures. However, there are still challenges that limit their development and application. Meanwhile, due to advancements in synthetic biology and protein engineering, we now have access to an array of more powerful tools [47,102,103]. This expanding toolbox provides us with numerous opportunities to explore novel hydrogel designs that offer superior mechanical properties, biological functionality, and environmental responsiveness. This section discusses current challenges as well as future opportunities of protein-based hydrogels (Figure 4).

### 4.1. Synthetic Biology for Material Production and Protein Hydrogel Innovations

Tailored hydrogels made of engineered proteins used to be limited by microbial production of sufficient proteins at large scales. This issue has mostly been solved by recent synthetic biology strategies as discussed in Section 3.2.1. Several engineered material proteins were produced at extremely high titers, even when their amino acid sequences are highly repetitive [67,68]. Development in non-canonical amino acid incorporation has further expanded the functional groups of amino acid sidechains, which can add new chemical functions to protein hydrogels [4,104,105]. In addition, advances in synthetic biology have greatly enhanced the synthetic power of microbial hosts [106,107,108,109], allowing complex biosynthetic pathways to be precisely controlled [110,111,112]. Many of these strategies can be used to produce protein materials in large scales to support hydrogel applications [64]. These strategies can also be used to yield proteins with various types of modifications, further diversifying functions and applications of protein hydrogels.

### 4.2. Sequence–Structure–Function Relationships

With increasing power on microbial synthesis of material proteins, protein-based hydrogels became limited by the ability to design hydrogels with predictable properties. To achieve this, it is important to fully understand the relationships between protein sequence, hydrogel structure, and hydrogel properties and functions. Such relationships can be learned by synthesizing a large number of proteins and by characterizing their corresponding hydrogels [113]. Furthermore, molecular and mechanical modeling approaches can be used to understand such relationships [48]. Once the relationships are fully understood, hydrogels with desirable properties, functionalities, and environmental responsiveness can be accurately designed and prepared.

### 4.3. Lack of Anisotropy in Protein Hydrogels

Most current hydrogels have uniformly dispersed protein networks and homogenous mechanical properties along all directions. On the other hand, some natural tissues have aligned protein fibers and exhibit anisotropic mechanical behavior that are critical to their functions [48]. Hence, it is crucial to develop strategies to fabricate structurally anisotropic protein hydrogels. One approach to create anisotropic hydrogel is to use pre-designed templates, such as microfluidic channels, to guide the alignment of protein building blocks during hydrogel formation [114]. This method has shown promising results in creating well-aligned fibers in hydrogels with controllable sizes and shapes. Another approach is to employ shear and diffusive forces simultaneously to generate cylindrical double-network hydrogels with both axially and radially aligned polymer fibers [115]. Additionally, proteins can be aligned to electric fields via their charged functional groups without altering their secondary structure [116]. Based on this principle, layered anisotropic hydrogels composed of soy protein isolate [117] and silk protein nanofibers [118,119] have been generated using electric fields to drive their hierarchical alignment by balancing their electrostatic interactions. This approach can be combined with other methods such as templated self-assembly, microfluidics, free-injection methods, and double-network strategy [120]. While these strategies to create anisotropic materials are promising, protein hydrogels produced from most methods thus far display relative weak mechanical properties [120] Thus, new strategies to fabricate anisotropic hydrogels with strong mechanical behaviors are needed.

### 4.4. Dynamic Cell–Hydrogel Interactions

Another limitation of current hydrogels is the lack of dynamic cell interactions. When using hydrogels as artificial ECMs, their mechanical and biological properties have profound influences on cells behaviors [48]. In natural systems, cells dynamically modify the mechanical and biological properties of their surrounding environment through processes such as matrix remodeling and contraction [121]. The changing ECM properties, in turn, influence cell behavior and function, leading to changes in tissue development and healing. However, current artificial ECMs either do not respond to cellular modifications as natural ECM or they change at drastically different scales. It is thus essential to engineer dynamic hydrogels that offer two-way interactions with cells and to fully understand these complex interactions for more advanced tissue-engineering applications.

### 4.5. Computation-Assisted Approaches

Computation-assisted approaches have become increasingly popular for the prediction of protein structures and can be potentially used to design protein hydrogels. Previous studies mostly focused on atomic and coarse-grained models. The former (such as RosettaDesign) relies on energy optimization and provides insights on interactions between functional groups [122]; the latter uses beads to represent groups of atoms in a protein to reduce complexity [123]. These two models focus on angstrom-to-micrometer-length scales and can provide complementary understanding of protein structures and interactions. However, both methods have limits when modeling protein hydrogels, where problems (e.g., defects, cracks) often exist at a higher-length scale. In recent years, artificial intelligence (AI)-empowered protein structure prediction and design have drastically transformed the field of structural biology [124,125]. Several successful examples of this approach have been reported for the generation of protein sequence with given structural elements [126,127] or iterative structural simulation [128,129]. AI-empowered direct protein sequence generation that bypasses structural features has also been developed [130,131] and could be a powerful tool in de novo design of protein hydrogels. Overall, the future of computational protein hydrogel design may appear as a popular approach.

## 5. Conclusions

Protein hydrogels have demonstrated many useful biomedical applications. With the advances in protein engineering, synthetic biology, and material engineering, future protein hydrogels will offer a combination of superior mechanical properties, biological functionality, and environmental responsiveness. The expanding toolboxes will not only allow us to better understand the sequence–property relationships, but will also create new opportunities for novel hydrogels. Moreover, AI-driven hydrogel design informed from micro- and mesoscale levels will accelerate the engineering process. We anticipate that these technologies will produce numerous protein hydrogels for diverse biomedical applications.

## Figures and Tables

**Table 1:** Advantages and disadvantages of hydrogels made by physical and chemical crosslinking methods.

Hydrogels	Advantage	Disadvantage	References
pH and ion-induced protein hydrogels	- mechanical properties can be adjusted by modifying the pH, protein concentration, and ionic strength of the solution - tunable crosslinking density, degradation profile, and controlled release - reversible	- susceptible to alterations in environmental conditions such as pH and ion concentration changes	[4,16,17,18,54,55]
Temperature-induced hydrogel formation	- mechanical properties can be adjusted by modifying the temperature and protein concentration - reversible	- susceptible to temperature alterations	[23,25,26]
Protein–protein interaction-based protein hydrogels	- simple mixing of two protein components without the use of harsh chemicals/buffers - tunable viscoelasticity - exhibit shear-thinning and self-healing properties	- short-term degradation	[31]
Light-controlled protein hydrogel	- crosslinking is initiated by UV with photo initiator - controllable crosslinking degree - precise spatial and temporal control - tunable mechanical properties	- toxicity of unreacted chemical and side products - ligand required for assembly might diffuse away in vivo, making reversible assembly challenging - crosslinking involving specific amino acids in the repeated sequences might compromise its functionality	[38,56,57]
Chemical crosslinker-based protein hydrogel	- exhibits elastic behavior - withstands axial deformation, stress, and mechanical corrosion - stable in wide range of conditions, including pH, ion concentration, temperature	- toxicity of unreacted chemical and side products - crosslinking involving specific amino acids in the repeated sequences might compromise its functionality	[45,58,59]
Enzymatic crosslinked hydrogels	- gelation kinetics can be controlled by adjusting the enzyme concentrations and activity - less toxic	- crosslinking involving specific amino acids in the repeated sequences might compromise its functionality	[49,51,52]

## Data Availability

Not applicable.

## Sample Availability

Not available.
